# Long-term contamination by non-native fish assemblages in a Neotropical floodplain

**DOI:** 10.1371/journal.pone.0311018

**Published:** 2024-11-11

**Authors:** Luis Artur Valões Bezerra, Simone Libralato, Jan Kubečka, Andre Andrian Padial

**Affiliations:** 1 Biology Centre of the Czech Academy of Sciences (BC-CAS), Institute of Hydrobiology, České Budejovice, Czechia; 2 Laboratorio de Análise e Síntese em Biodiversidade (LASB), Departamento de Botânica, Programa de Pós-Graduação em Ecologia e Conservação (PPGECO-UFPR) and Programa de Pós-Graduação em Botânica, Universidade Federal do Paraná, Curitiba, Brazil; 3 National Institute of Oceanography and Applied Geophysics—OGS, Trieste, Italy; 4 Programa de Pós-Graduação em Ecologia de Ambientes Aquáticos Continentais, Núcleo de Pesquisa em Limnologia, Ictiologia e Aquicultura (NUPELIA), Universidade Estadual de Maringá, Maringá, Brazil; University of Florida, UNITED STATES OF AMERICA

## Abstract

Biological invasions are a major threat to biodiversity in species-rich regions. Therefore, it is important to understand mechanisms behind the long-term establishment of non-native fish species in aquatic environments in the Neotropical region. Here, we associated fish biomass, species richness, and the proportion of non-native species (contamination and Kempton’s indices) to quantify the non-native pressure over fish biodiversity in lakes and rivers of the Parana River floodplain, seasonally, from 2000 to 2017. We divided species into native and non-native assemblages sampled in spatio-temporal gradients. Temporal trends were examined using linear regressions and generalised additive models. Fish biomass in gillnets increased for both native and non-native fish species, but their Kempton indices were inversely correlated. Extinction of native species occurred locally with biotic differentiation of non-native species in lakes, rivers, and ecosystem contamination. A constant increase in fish biomass resulted in overwhelming biodiversity of non-natives at the end of the time series evaluated. Native biotic resistance to introductions was not detected in deterministic trends. The observed patterns were consistent with previous studies showing native biotic homogenisation and extinction of species in response to biological invasions, landscape fragmentation, and riverine impoundments. Increases in abundance and species richness of non-native fish were the biodiversity drivers that resulted in non-native species outweighing native species in the Parana floodplain.

## Introduction

The stabilizing effect of species diversity on ecosystem properties [1, 2] has been investigated particularly from the conception of the diversity-stability hypothesis [3, 4], translating the association between biomass and species richness a proxy of biodiversity, the last stabilizing with the total community biomass and productivity increases [4]. However, biomass and biodiversity relationships can be more abstruse in real-world systems [5] because biomass and species richness are not always related in natural environments [6, 7]. For instance, biomass-diversity relationships can be under scrutiny in ecosystems under permanent influence of biological invasions and propagule pressure [8], since the asynchrony in the abundance of individual populations in response to the environment and biotic interactions not always promotes the coexistence [9].

In the context of biological invasions, the biomass of a non-native population represents the number of dispersals and individual growth, and the species richness at each site represents their local colonisation pressure on the species pool [10]. Biotic acceptance occurs whenever the colonisation pressure increases in association with an increase in the number of native species [11]. Among negative interactions, species translocated and adapted to a new environment outside their native range can have unpredictable negative effects [12, 13] that varies from a few species dominating complex communities [14] to massive loss of biodiversity [15], and increased extinction rates [13, 16]. Indeed, the effects of two non-native species may be greater than the sum of the individual effects of each species if they are interacting with cascading ecosystem effects such as eutrophication [17, 18], which can be worse in highly diverse environments, such as South American lakes and rivers [19].

Long-term research programs in ecosystems under strong propagule pressure are an opportunity to test for the relationship between biomass and species richness in the context of biological invasions [20]. A temporal or spatial increase in biomass represents the growth [7] or multiplication [21] of individuals per species and species per area [22]. A correlated increase of biomass and species richness in species-rich communities also represents a scenario of biotic differentiation [23]. The winner introduced assemblage gain space and niche-mediated processes can lead to the extinction of losers [14], supposedly decreasing the stability [9] of the whole system. Negative associations between biomass and species richness represent extinction pressures in two ways: 1) local species loss with positive effects on overall biomass [24], e.g., loss of rare species; or 2) species richness increases negatively affecting biomass, e.g., when new species compete and hamper the development of common and dominant species [6].

The floodplain of the upper Parana River is regularly monitored and faces biological invasions after the damming of the rivers by the Itaipu (1982) and Porto Primavera (1997) dams, especially because a strong barrier (the waterfalls of Sete Quedas) was inundated by the Itaipu reservoir [25, 26]. As more species have been introduced and adapted to rivers [27, 28] and reservoirs [29], the introduction of non-native fish into sections free of dams has increased [30]. Recent investigations on fish communities have highlighted the regulation of flooding in reservoirs influencing fish reproduction [31], recruitment [32], and metacommunities [33]. Nearby fish farms are another source of non-native fish entering floodplain rivers and lakes [34]. Fish are also used as bait, migrate from newly created reservoirs, and are intentionally released for other purposes [35, 36].

We examined biodiversity patterns of native and non-native fish species at nine localities (three rivers and six lakes) in the upper Parana floodplain sampled regularly from 2000 to 2017 as part of a long-term ecological research (LTER) program. We measured the correlations of biomass and species richness and contamination of lakes and rivers by non-native species across space and time. We expected a high correlation of the overall biomass and species richness trough time and space under influence of the”insurance hypothesis” [37]. In the long term, we expected non-native diversification in contaminated environments because of biotic acceptance [37], and a loss of native species and biotic homogenisation in the lakes and rivers studied [31, 33, 38, 39]. In addition, we expected that a loss of native biodiversity would decrease the invasion resistance, thus promoting the establishment of non-native species [40], represented by the increasing correlation of biomass and species richness of non-native populations through time.

## Methods

### Study site and sampling

The staff of the LTER of the Universidade Estadual de Maringá (identified in Brazil as PELD/CNPq/UEM/site 6, Brazil) seasonally sampled three riverine basins (“Baia”, “Ivinheima”, and “Paraná”) of the upper Paraná River floodplain. Fish assemblages were captured both by gillnetting (lakes and rivers, from 2000 to 2017) and beach seining (lakes, from 2003 to 2017) in the floodplain. The LTER was carried out in accordance with internationally accepted practices for fish monitoring and regulated by the “Comissão de Ética no Uso de Animais”, the CEUA-UEM ethics committee). No killings were needed to perform our comparisons beyond that promoted to feed the LTER. No permits were required for the described study, which complied with all relevant regulations.

We selected nine localities (six lakes, namely: “Garças”, “Guarana”, “Fechada”, “Patos”, “Pau Veio”, and “Ventura”; and the channels of three main rivers of the above-mentioned basins) monitored from 2000 to 2017 as representative of different floodplain habitats. Standardized monitoring occurred in the following sampling sites: Lakes “lfec” (‘Fechada’) “lgar” (‘Garças’), “lgua” (‘Guarana’), “lpat” (‘Patos’), “lpve” (‘Pau Veio’), “lven" (‘Ventura’) and rivers “rbai” (‘Baia’), “rivi” (‘Ivinheima’), “rpar” (‘Paraná’).

Gillnetting was always conducted for 24h in GPS referenced localities. Each gillnet set was 220 m long in a combination of meshes (2.4, 3, 4, 5, 6, 7, 8, 10, 12, 14, and 16 cm between knots), resulting in 368 m^2^ of effort per locality (S1 Table). The seine net had a length of 20 m (0.5 cm between knots) and the sampled area varied from 30.2 m² to 367.09 m², according to the sampled biotope (average seined area 247.69 ± 51.52 m^2^). Further details of the standardised sampling program are available in the literature [20, 24]. In each assemblage, we identified native and non-native fish species (S2 Table) [41].

### Biomass, species richness, contamination index, and Kempton`s Biodiversity Index (*Q*)

In each locality and campaign, a sampling unit (*s*) represented a single gillnetting or seining event. For each species (*j*) in *s*, we calculated the biomass (*B*_*j*_) from the weight of all individuals (*ω_i_*, g) per area (*A*, in m^2^):

$$B_{j}=\frac{\sum_{i=1}^{N_{j}}\omega_{ij}}{A},$$ (Eq 1)

where *N*_*j*_ is the number of individuals for each *j*-th species. The sum of *B*_*j*_ per sampling unit (*B*_*s*_) and species richness were calculated for native and non-native assemblages at each *s*. We averaged *B*_*s*_ and species richness per locality when evaluating temporal variation, or per campaign when evaluating spatial trends in each locality.

The proportion of non-native species at each *s* represented the assemblages’ Contamination Index *CI*_*r*_ [42], measured as the number of non-native species (*R*_*nn*_) over the total species richness (natives and non-native, *R*_*n*_ and *R*_*nn*_, respectively):

$$CI_{r}=\frac{R_{nn}}{R_{nn}+R_{n}}$$ (Eq 2)

The same was recalculated with the biomass of native (*B*_*n*_) and non-native (*B*_*nn*_) assemblages, representing a biomass contamination index (*CI*_*b*_). Since the effect of two non-native species could be greater than the sum of the two parts, we calculated different metrics associating native and non-native biomass and species richness in additive and multiplicative forms.

We also measured the association between biomass and species richness as a proxy of biodiversity [2, 4] at each *s*, this is, the multiplicative interaction between species richness and biomass of all non-native species (*R*_*nn*_ *x B*_*nn*_) over the total biodiversity (*R*_*nn*_ *x B*_*nn*_ +*R*_*n*_ × *B*_*n*_), the “ecosystem contamination index” (*CI*_*e*_):

$$CI_{e}=\frac{R_{nn}xB_{nn}}{\left(R_{nn}xB_{nn}+R_{n}\times B_{n}\right)}$$ (Eq 3)

In addition, we averaged biomass and species richness in different ways, based on:

$$CI_{a}=\left(CI_{b}+CI_{r}\right)/2,\;\text{and}$$ (Eq 4)

$$CI_{sq}=\sqrt{CI_{b}\times CI_{r}},$$ (Eq 5)

considering previous indexes, resulting in the comparison of five indexes (*CI*_*a*_, *CI*_*b*_, *CI*_*e*_, *CI*_*r*_, *CI*_*sq*_).

We evaluated Spearman’s *rho* values between biomass and species richness to represent their joint effect per campaign and locality, which could be correlated positively, inversely (negative), or not related (nearly zero). Finally, we measured the Kempton’s index (*Q*) to represent biodiversity [43] at each campaign, considering the species richness (*R*), and biomass (*B*) of native or non-native individuals in k localities through time:

$$Q=\sum_{k=1}^{k}\left(\frac{R}{2\times \log\;e\left(\frac{B_{4}}{B_{2}}\right)}\right)\times \frac{1}{k},$$ (Eq 6)

where *B*_4_ and *B*_2_ were the upper and lower quartiles of the biomass distribution (respectively 75% and 25% of the data). The *Q* index averaged across localities served as a validation of contamination indices (*CI*) and *rho* correlations through time.

### Statistical analyses

All analyses were performed in R [44] and data manipulation and plots in the “tidyverse” package [45]. When data distribution was uneven across campaigns (Shapiro-Wilks-lambda, *P* < 0.05), we used Generalized Additive Models (GAM) and the “gam” function of the “mgcv” package [46] with an Auto Regressive (AR) corelation pattern (corAR1, “nlme” package) [47]. We predicted biomassand species richness as response variables (Rs) to fish origin and time as explanatory variables (Rs ~ Origin * Time) plus localities as random effects at the significance level of α < 0.05. The seined biomass was log10 transformed by log (x + 1) to decrease the influence of outliers in the model.

The *CI*, *Q*, *rho* were response variables to sampling method and time (Method * Time) in GAM models with corAR1 and random effects. Finally, we explained the temporal variation of individual Rs in each locality. We evaluated effective degrees of freedom (edf) to assess the statistical significance in penalized GAM models, and reference degrees of freedom (Ref.df) in the calculation of parametric tests [46]. If the adjusted R^2^ (adj-R^2^) was small, the strength of statistically significant trends were identified by the edf [48]. Interactions between explanatory variables (Time*Method and Time*Origin) were not tested for the GAM models including *CI*, *Q*, and *rho* as response variables because interactions inflated modelled degrees of freedom and increased chances of Type II errors.

We compared the distribution of *rho* values between native and non-native species with the Wilcoxon’s test [49] across localities and campaigns. For native *vs*. non-native comparisons, we did not discriminate assemblages between gillnetting and seining (also, we did not calculate *Q* and *rho* in each campaign and locality through time) because sampling methods did not influence the variability of biodiversity metrics across localities (S1 Fig).

## Results

### Seasonal variation and propagule pressure

**Fig 1:**
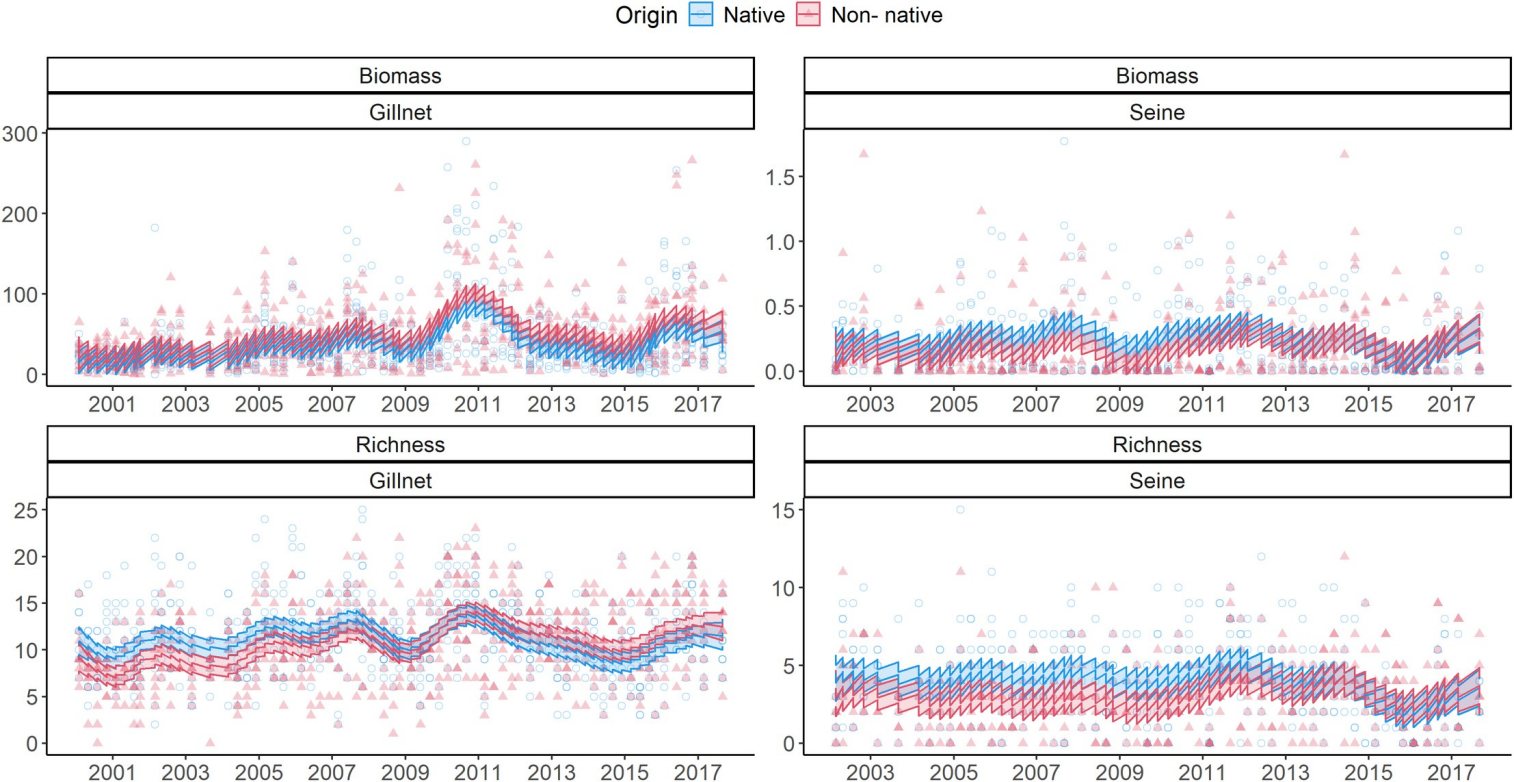
Species richness and biomass (g.m^-2^) of native (blue) and non-native (red) species by seasonal sampling campaigns and methods (gillnet and seine) in the Paraná River floodplain from 2000 to 2017. The seined biomass is in log10 scale. Lines represent Generalized Additive Models (GAM, with an autoregressive correlation pattern), explained by origin * time and localities as random effects, with *P* < 0.05 (see Table 1).

The sampling effort registered 157 species (85 native, 68 non-natives, and four not categorized; S2 Table). Overall, non-native species accounted for 43% of the species pool and at the beginning of the time series (year 2000; non-native to native species richness ratio of 26:42 species). At the end, the number was closer to the 1:1 ratio (46:50 in 2015, 47:52 in 2016, and 39:47 in 2017). Time series seasonal fluctuations of native and non-native species were correlated, pointing to the influence of positive interactions or external factors shaping the biomass and species richness time series (*e*.*g*., water level; Fig 1).

Biomass and species richness escalated through time independently of the species origin (native vs non-native; Table 1) but depending on the sampling methods: gillnet explained 23% of the captured biomass (Adj-R^2^ = 0.23; edf = 15.64; *P* < 0.01) and 16% of the species richness (Adj-R^2^ = 0.16; edf = 16.48; *P* < 0.01) increases. In gillnets, biomass and species richness resulted in a higher dominance of non-natives at the end of the time series. Contrastingly, in seines, average biomass (edf = 14.02; *F* = 3.41; *P* = 0.133) and species richness (edf = 14.02; *F* = 3.41; *P* = 0.133) were constant for non-native assemblages across sampling periods. In addition, seine captures reflected a decrease in the coefficient of variation of biomass and species richness (funnel-shape trough time).

**Table 1:** Generalised Additive Models (GAM) with an autoregressive correlation pattern (*Phi*) of fish biomass and species richness (response variables) to the interaction between (fish origin and time), and sampled localities as explanatory random effects, captured in the Parana River floodplain from 2000 to 2017 in 68 gillnetting and 60 seining events (2003–2017).

Method	Origin	Response	edf	Ref.df	*F*	*P*	Adj-R^2^
Gillnet	Native	Biomass	15.64	16.77	21.15	< 0.01	0.23
Gillnet	Non- native					< 0.01	
Seine	Native		14.02	15.99	3.41	0.022	0.08
Seine	Non- native					0.133	
Gillnet	Native	Richness	15.87	16.84	12.42	< 0.01	0.16
Gillnet	Non- native					< 0.01	
Seine	Native		13.62	15.73	4.28	< 0.01	0.10
Seine	Non- native					0.108	

Adjusted R^2^ (Adj-R^2^), effective degrees of freedom (edf, and reference Ref.edf), Fisher’s F, and *P*–values are represented.

**Fig 2:**
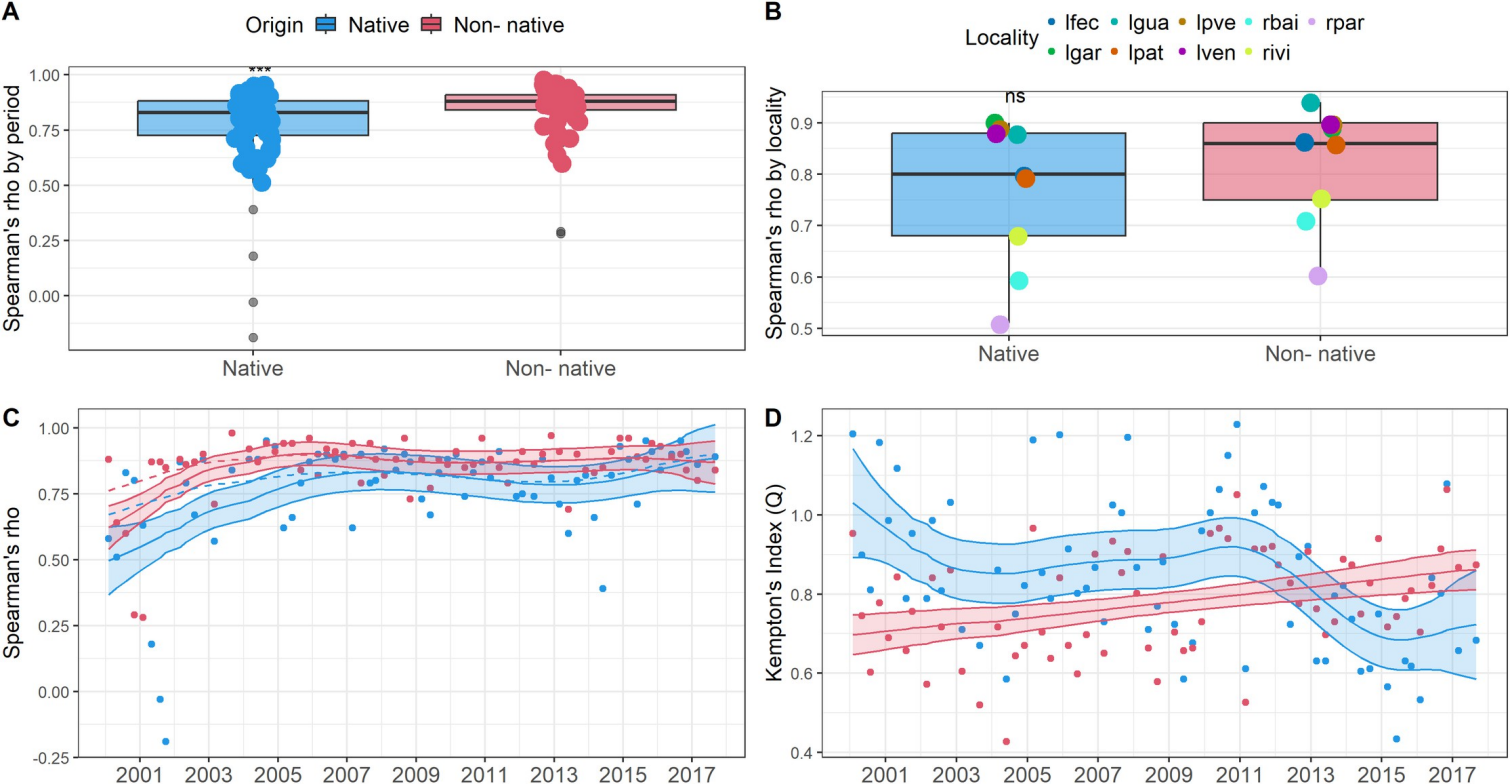
Spearman’s correlation coefficient (*rho*) and Kempton’s Index (*Q*) between biomass (g.m^-2^, gillnetting and beach seining in lakes, see Methods section for further details) and species richness of native and non-native fish at six lakes and three riverine channels of the Paraná River floodplain sampled seasonally from 2000 to 2017. Frames **A** and **B** represent the comparison of *rho* by sampling periods and localities; **C** and **D** are the long-term *rho* and *Q* trends in Generalized Additive Models (GAM, with an autoregressive correlation pattern), explained yb time with *P* < 0.05. Differences without considering outliers (***) and non-significative (ns) comparisons are also identified in **A** and **B**. Localities are represented in **B** with “l” standing for lakes and “r” for rivers (lfec, lgar, lgua, lpat, lpve, lve, rbai, rivi, and rpar). Lakes were “Garças”, “Guarana”, “Fechada”, “Patos”, “Pau Veio”, and “Ventura”; and the rivers are “Baia”, “Ivinheima”, and “Paraná”. Grey dots in A represent outliers, and dashed lines in C represent the trend after the elimination of outliers.

Median *rho* (biomass species richness correlations) values were higher than 70% for both assemblages over time (Fig 2A), except for two campaigns in which we observed a high type I error (α > 0.05) with tails that prevented the quantification of *P*-values. The greatest *rho* occurred for non-natives at the “Guarana” (‘lgua’) lake. The distribution of *rho* values across localities did not differ between the native and non-native assemblages (Fig 2B).

The increase of *rho* values through time represented a biodiversity increase, given positive interaction between biomass and species richness, but depending on fish origin (adj-R^2^ = 0.32; edf = 8.83; *F* = 8.39; *P* < 0.01). Despite increasing for both assemblages, *rho* values (without outliers) were higher for non-natives (Wilcox test, *W* = 1376.5; *P* < 0.01). Yet with loss of species, a pool of native species increased in the correlation of biomass and occurrence in recent years. Small or negative *rho* correlations appeared at the beginning of the time series (Fig 2C), with a late predominance of positive values. Negative *rho* values were not considered relevant, and the removal of outliers did not influence (adj-R^2^ = 0.25; edf = 5.82; *F* = 7.1; *P* < 0.01) the observed trends (Fig 2A and 2C).

The *Q* biodiversity trends were inversely proportional (Fig 2D), depending on fish origin (adj-R^2^ = 0.33; edf = 13.8; *F* = 8.32; *P* < 0.01). This trend was associated with a greater biomass variation, and an inverse relationship between native and non-native species through time, with a loss of *Q* biodiversity of native species at the end of the time series (adj-R^2^ = 0.246; edf = 4.75; *F* = 3.66; *P* < 0.01). The *Q* biodiversity of non-native species increased almost 25% (adj-R^2^ = 0.12; edf = 1; *F* = 9.98; *P* < 0.01), which validated the contamination by non-native species observed in each ecosystem, such as represented by the ecosystem contamination (*CI*_*e*_) in the following section.

### Contamination indices

**Fig 3:**
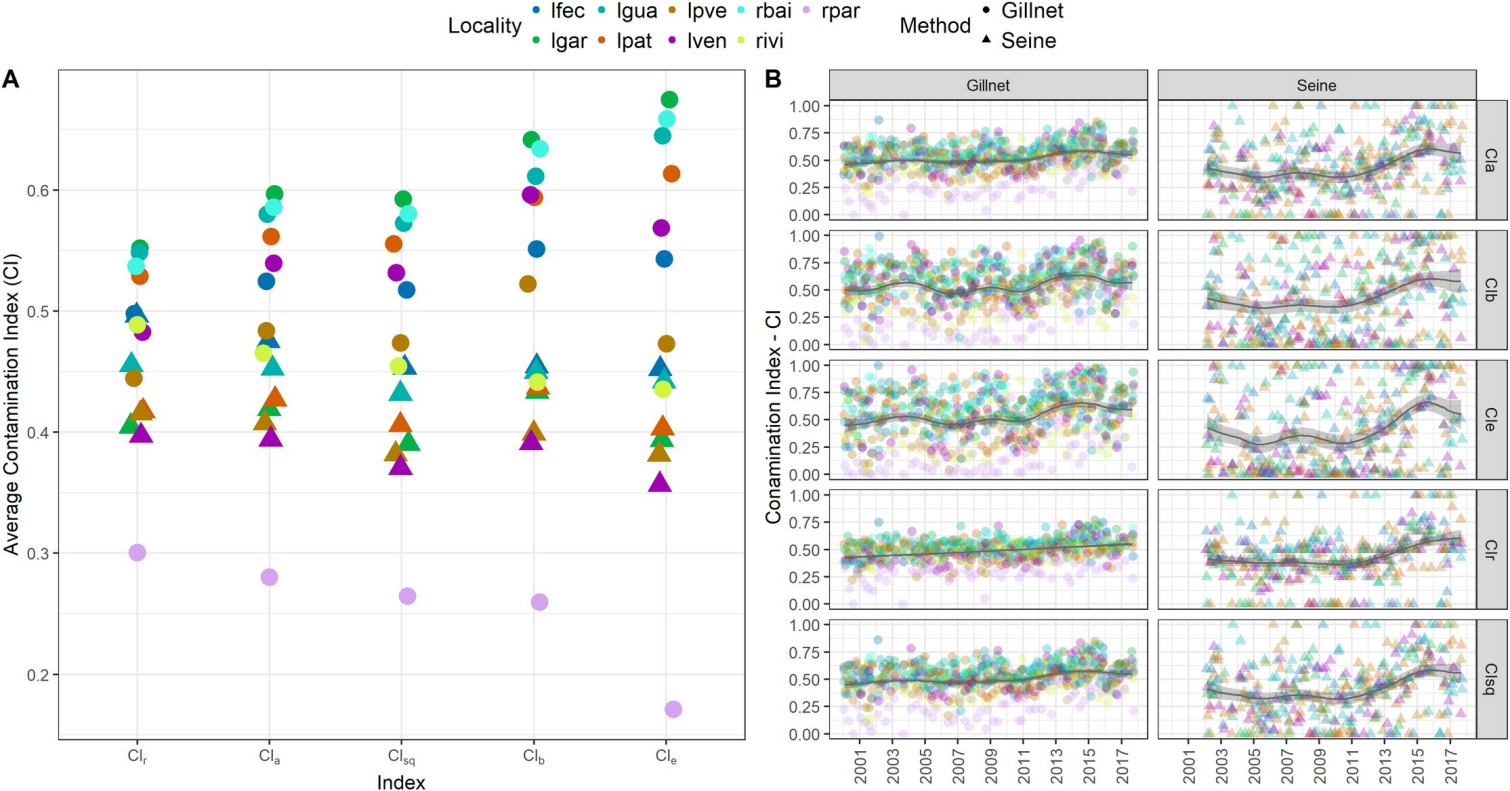
**A** ‐‐ Average Contamination Indexes (*CI*, 2000 to 2017) of fish assemblages in lakes and rivers of the Paraná River floodplain. The index accounted for the proportion of non-native species in the assemblages based on fish species richness (*CI*_*r*_), biomass (*CI*_*b*_), as well as richness and biomass (*CI*_*e*_), in additive (*CI*_*a*_) and multiplicative (*CI*_*sq*_) forms (further details in the methods section). **B** ‐‐ Temporal variation of *CI* indices in each campaign. Sampled environments are represented with “l” standing for lakes and “r” for riverine channels (‘lfec’, ‘lgar’, ‘lgua’, ‘lpat’, ‘lpve’, ‘lve’, ‘rbai’, ‘rivi’, and ‘rpar’). Lakes were “Garças”, “Guarana”, “Fechada”, “Patos”, “Pau Veio”, and “Ventura”; and the rivers are “Baia”, “Ivinheima”, and “Paraná”. Lines represent Generalized Additive Models (GAM, with an autoregressive correlation pattern), explained by method * time, with *P* < 0.05, and localities as random effects.

The contamination level varied with sampling method, locality, and campaigns, but temporal trends (Fig 3) and the type of *CI* index did not vary across localities and campaigns, regardless a few exceptions like *CI*_*b*_ at ‘lven’, which was greater than ‘lpat’ inside the margin of standard deviations (Table 2). Overall contamination, such as reflected by each index, peaked at two lakes (‘lgar’ and ‘lgua’) and at a river (‘rbai’) sampled by gillnets. However, the variability of each index differed, depending on localities and methods: The *CI*_*e*_ provided the most informative patterns of contamination, especially if compared with additive (*CI*_*a*_) and species richness (*CI*_*r*_) indices, but also with those accounting for species richness and biomass (*CI*_*sq*_), and biomass *CI*_*b*_ separately. Overall, greater variance resulted from the inclusion of the interaction of biomass and species richness in the calculation of *CI*_*e*_, under influence of sampling tool in each locality.

**Table 2:** Average contamination indices (*CI*) ± standard deviation.

Locality	Method	CIe	CIb	CIa	CIsq	CIr
lgar	Gillnet	0.67 ± 0.16	0.64 ± 0.15	0.6 ± 0.1	0.59 ± 0.09	0.55 ± 0.07
rbai	Gillnet	0.66 ± 0.15	0.63 ± 0.12	0.59 ± 0.08	0.58 ± 0.08	0.54 ± 0.08
lgua	Gillnet	0.64 ± 0.18	0.61 ± 0.16	0.58 ± 0.1	0.57 ± 0.11	0.55 ± 0.07
lpat	Gillnet	0.61 ± 0.19	0.59 ± 0.17	0.56 ± 0.11	0.56 ± 0.1	0.53 ± 0.07
lven	Gillnet	0.57 ± 0.24	0.6 ± 0.2	0.54 ± 0.14	0.53 ± 0.13	0.48 ± 0.1
lfec	Gillnet	0.54 ± 0.17	0.55 ± 0.16	0.52 ± 0.1	0.52 ± 0.1	0.5 ± 0.09
lpve	Gillnet	0.47 ± 0.21	0.52 ± 0.18	0.48 ± 0.11	0.47 ± 0.11	0.44 ± 0.09
lfec	Seine	0.45 ± 0.32	0.45 ± 0.29	0.48 ± 0.22	0.45 ± 0.24	0.5 ± 0.19
rivi	Gillnet	0.44 ± 0.21	0.44 ± 0.18	0.47 ± 0.11	0.45 ± 0.12	0.49 ± 0.08
lgua	Seine	0.44 ± 0.38	0.45 ± 0.37	0.45 ± 0.3	0.43 ± 0.3	0.46 ± 0.26
lpat	Seine	0.4 ± 0.34	0.44 ± 0.33	0.43 ± 0.25	0.41 ± 0.26	0.42 ± 0.22
lgar	Seine	0.39 ± 0.37	0.43 ± 0.36	0.42 ± 0.27	0.39 ± 0.28	0.4 ± 0.24
lpve	Seine	0.38 ± 0.4	0.4 ± 0.38	0.41 ± 0.31	0.38 ± 0.32	0.42 ± 0.27
lven	Seine	0.36 ± 0.35	0.39 ± 0.33	0.39 ± 0.25	0.37 ± 0.26	0.4 ± 0.21
rpar	Gillnet	0.17 ± 0.17	0.26 ± 0.2	0.28 ± 0.14	0.26 ± 0.14	0.3 ± 0.1

The based were based on fish species richness (*CI*_*r*_), biomass (*CI*_*b*_), as well as richness and biomass (*CI*_*e*_), in additive (*CI*_*a*_) and multiplicative (*CI*_*sq*_) forms in localities of the Parana River floodplain, from 2000 to 2017 (further details in the Methods section). Localities are represented with “l” standing for lakes and “r” for riverine channels (‘lfec’, ‘lgar’, ‘lgua’, ‘lpat’, ‘lpve’, ‘lve’, ‘rbai’, ‘rivi’, and ‘rpar’). Lakes were “Garças”, “Guarana”, “Fechada”, “Patos”, “Pau Veio”, and “Ventura”; and the rivers are “Baia”, “Ivinheima”, and “Paraná”. The Table was ordered by *CI*_*e*_ values.

There were greater *CI* contamination slopes and model explanation (adjusted R^2^) for gillnet assemblages (estimated slope *Beta*, Table 3) than in seines. The *CI* median contamination value was above 60% for assemblages captured by gillnets in environments outside protected areas (‘lgua’ and ‘rbai’), and 3.9 times higher than the least contaminated environment (Paraná River; ‘rpar’). Paired differences occurred particularly between riverine channels and lacustrine environments (highlighting ‘rpar’ *vs*. ‘lgar’), but also between rivers (e.g., ‘rpar’ *vs*. ‘rbai’).

**Table 3:** Results of Generalized Additive Models (GAM, with an autoregressive correlation pattern and localities as random effects) of contamination indices *CI*.

Method	Index	*Beta* ± sd	edf	Ref.df	*F*	*P*	Adj-R^2^
Gillnet	CIsq	0.62 ± 0.01	7.48	9.24	8.11	< 0.01	0.27
Gillnet	CIa	0.63 ± 0.01	7.77	9.58	7.87	< 0.01	0.26
Gillnet	CIr	0.57 ± 0.01	1.31	1.55	57.19	< 0.01	0.26
Gillnet	CIe	0.72 ± 0.02	9.06	11.08	6.75	< 0.01	0.25
Gillnet	CIb	0.68 ± 0.02	9.79	11.90	5.06	< 0.01	0.21
Seine	CIe	0.43 ± 0.03	5.89	7.32	6.85	< 0.01	0.13
Seine	CIa	0.46 ± 0.03	5.25	6.52	7.07	< 0.01	0.13
Seine	CIsq	0.43 ± 0.03	4.97	6.18	7.28	< 0.01	0.12
Seine	CIr	0.47 ± 0.02	3.95	4.93	8.79	< 0.01	0.12
Seine	CIb	0.44 ± 0.03	4.28	5.33	5.97	< 0.01	0.09

Trends across campaigns are shown in Fig 3B. Adjusted R^2^ (Adj-R^2^), effective degrees of freedom (edf, and reference Ref.edf), Fisher’s F, *P*–values, and the estimated slope (*Beta* ± sd) are represented.

**Fig 4:**
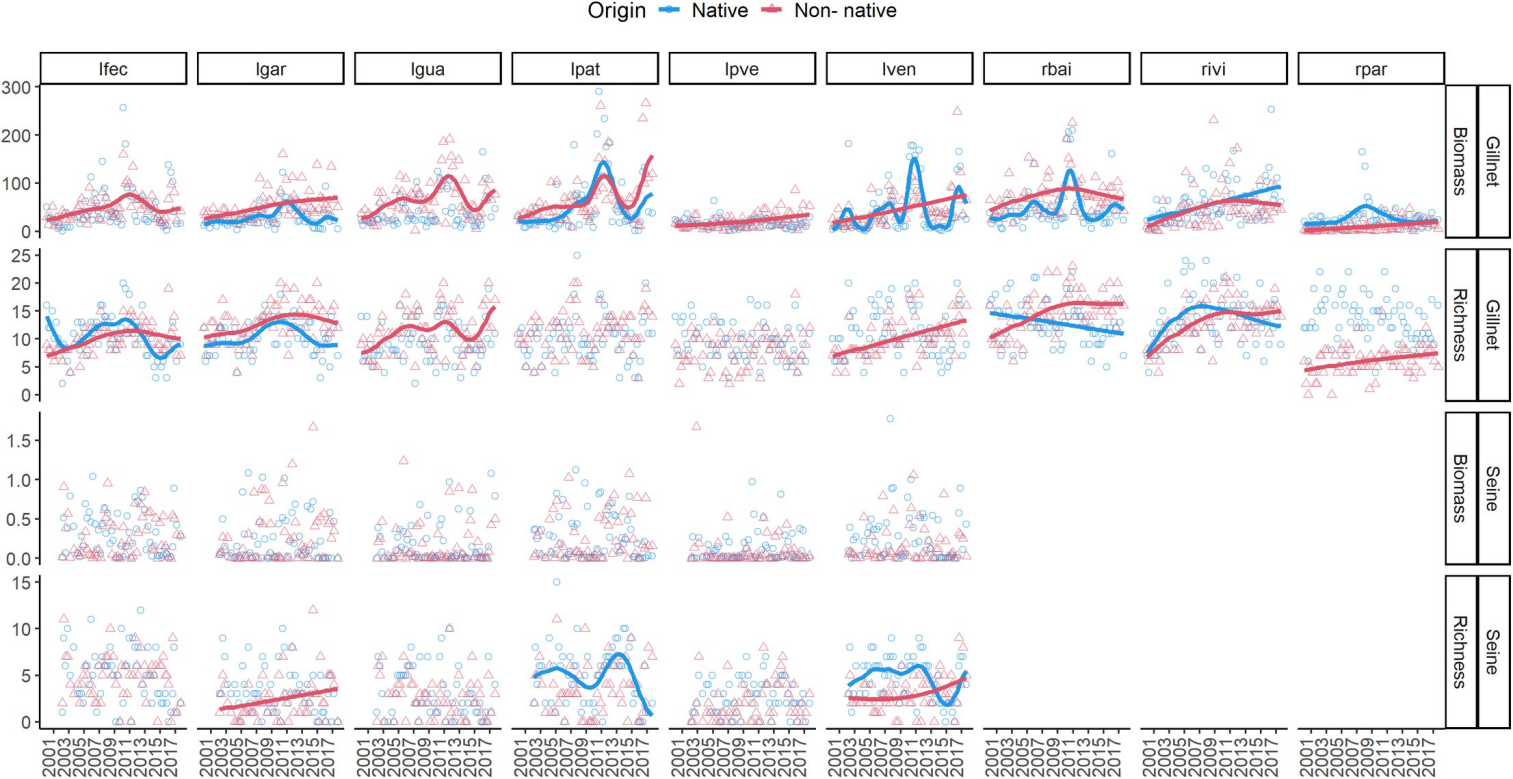
Native (blue circles) and non-native (red triangles) fish assemblage`s biomass and species richness in gillnets and seines, from 2000 to 2017, in the Paraná River floodplain. The strip labels represent sampled environments, with “l” standing for lakes and “r” for riverine channels (‘lfec’, ‘lgar’, ‘lgua’, ‘lpat’, ‘lpve’, ‘lve’, ‘rbai’, ‘rivi’, and ‘rpar’). Lakes are “Garças”, “Guarana”, “Fechada”, “Patos”, “Pau Veio”, and “Ventura”; and the rivers are “Baia”, “Ivinheima”, and “Paraná”. Lines represent Generalized Additive Models (GAM, with an autoregressive correlation pattern). Trendlines are only included for the relationships when *P* < 0.05.

Following overall trends, most of the biomass and species richness variation in each locality was explained by individuals captured in gillnets. The greatest native to non-native ratio of biomass and species richness occurred in “rpar” in comparison with other localities during the whole sampling period (Fig 4 - rpar). Non-native biomass and species richness increased through time at the most contaminated lagoons ‘lgar’ and at ‘lgua’ (Table 4). However, the steepest increase occurred in the most contaminated river ‘rbai’ (*Beta* = 74.09 ± 4.5), with and increase in the biomass of non-native species and loss of native species.

**Table 4:** Results of Generalized Additive Models (GAM, with an autoregressive correlation pattern) of biomass and species richness as response variables to time.

Method	Localities	Origin	Response	*Beta ± sd*	edf	Ref.df	*F*	*P*	Adj-R^2^
Gillnet	lfec	Non- native	Biomass	48.42 ± 2.99	5.31	6.60	4.06	<0.01	0.29
Gillnet	lgar	Non- native	Biomass	51.48 ± 3.42	1.68	2.09	7.49	<0.01	0.18
Gillnet	lgar	Native	Biomass	29.55 ± 2.14	8.32	10.23	3.82	<0.01	0.38
Gillnet	lgua	Non- native	Biomass	65.99 ± 4.05	6.92	8.57	4.32	<0.01	0.35
Gillnet	lpat	Non- native	Biomass	66.57 ± 5.2	7.06	8.73	4.34	<0.01	0.36
Gillnet	lpat	Native	Biomass	55.75 ± 5.64	7.53	9.30	5.12	<0.01	0.42
Gillnet	lpve	Non- native	Biomass	20.94 ± 1.71	1.50	1.85	9.88	<0.01	0.19
Gillnet	lven	Non- native	Biomass	46.12 ± 4.07	1.00	1.00	17.07	<0.01	0.19
Gillnet	lven	Native	Biomass	44.56 ± 4.21	12.54	14.66	6.12	<0.01	0.57
Gillnet	rbai	Non- native	Biomass	74.09 ± 4.5	2.43	3.03	2.89	0.04	0.11
Gillnet	rbai	Native	Biomass	48.68 ± 4.36	9.81	11.91	3.38	<0.01	0.39
Gillnet	rivi	Non- native	Biomass	48.48 ± 4.56	2.25	2.81	4.84	<0.01	0.16
Gillnet	rivi	Native	Biomass	58.56 ± 4.63	1.00	1.00	18.88	<0.01	0.21
Gillnet	rpar	Non- native	Biomass	10.31 ± 1.2	1.00	1.00	18.13	<0.01	0.20
Gillnet	rpar	Native	Biomass	27.56 ± 2.65	5.12	6.38	3.47	<0.01	0.25
Gillnet	lfec	Non- native	Richness	10 ± 0.29	2.80	3.49	6.81	<0.01	0.26
Gillnet	lfec	Native	Richness	10.47 ± 0.35	6.71	8.32	5.35	<0.01	0.40
Gillnet	lgar	Non- native	Richness	12.75 ± 0.36	2.87	3.59	4.55	<0.01	0.19
Gillnet	lgar	Native	Richness	10.41 ± 0.31	4.69	5.85	4.79	<0.01	0.29
Gillnet	lgua	Non- native	Richness	11.22 ± 0.42	5.64	7.02	3.06	0.01	0.24
Gillnet	lven	Non- native	Richness	10.19 ± 0.34	1.17	1.31	22.35	<0.01	0.31
Gillnet	rbai	Non- native	Richness	14.72 ± 0.36	2.58	3.23	9.92	<0.01	0.32
Gillnet	rivi	Native	Richness	13.66 ± 0.49	3.34	4.16	4.76	<0.01	0.22
Gillnet	rivi	Non- native	Richness	12.91 ± 0.35	3.07	3.83	12.48	<0.01	0.42
Gillnet	rpar	Non- native	Richness	6.07 ± 0.31	1.36	1.63	4.31	0.02	0.10
Seine	lpat	Native	Richness	4.9 ± 0.37	5.06	6.30	3.15	0.01	0.24
Seine	lven	Non- native	Richness	3 ± 0.23	2.00	2.50	3.90	0.02	0.13
Seine	lven	Native	Richness	4.62 ± 0.25	6.15	7.64	3.87	<0.01	0.33

Trends across campaigns (slope *Beta*) are shown in Fig 4. Adjusted R^2^ (Adj-R^2^), effective degrees of freedom (edf, and reference Ref.edf), Fisher’s F, *P*–values, and the estimated slope (*Beta* ± sd) are represented. The Table is ordered by method, response variable, and localities, respectively. Acronyms with “l” stand for lakes and with “r” for riverine channels (‘lfec’, ‘lgar’, ‘lgua’, ‘lpat’, ‘lpve’, ‘lve’, ‘rbai’, ‘rivi’, and ‘rpar’). Lakes are “Garças”, “Guarana”, “Fechada”, “Patos”, “Pau Veio”, and “Ventura”; and the rivers are “Baia”, “Ivinheima”, and “Paraná”.

In fact, environments with a relatively high *CI*_*e*_ (above 0.5, ‘lgar’, ‘lven’, ‘rbai’) were also those with the steepest increase in the number of non-native species through time. In gillnets, riverine environments such as ‘rpar’ had the smallest proportion of non-native species at the beginning of the time series, averaging five non-native species and 15 native species in 2000, then increasing to 6:14 in 2017, the highest proportional increase. Nevertheless, the native species richness did not variate through time in ‘rpar’ (βtime = -0.01; *F* = 1.6; df = 66; *P* = 0.11).

## Discussion

We investigated the association between biomass and species richness representing the biodiversity of fish assemblages in lakes and rivers in the Parana River floodplain which has seen growing presence of non-native species in the last decades. The results of *rho* correlations and the biomass inter-quartile range (measured as the denominator of *Q*) were consistent with the” insurance hypothesis”. In addition, our results were consistent with the increase of native and non-native biodiversity in recent periods, such as expected by “biotic acceptance”. Still, the loss of native species richness can be associated with negative interactions between native and non-native species, successful invasions, and the effect of dominant populations [7, 9, 50]. The increase of biomass and biomass variability of native and non-native species resembled a dominance of “a few winners over many losers” [14], in which a few dominant populations control fish captures and influence negatively the native species richness. However, biodiversity measures and the contamination indexes reflected increases either in biomass and non-native species richness, therefore the establishment of the non-native pool and biotic differentiation [43, 51].

The ongoing biotic differentiation of non-native fish assemblages and native biotic homogenisation is a trend in Brazilian aquatic ecosystems [21, 24, 52, 53]. Our findings agreed with previous studies showing the colonisation of the upper stretches of the river by at least 30 species after the last important damming events (downstream of the Itaipu reservoir, and upstream of the Porto Primavera reservoir) [26]. The resulting fish introductions could be associated with the loss of native species in recent years [28], characterizing a persistent impact, particularly in seined habitats of lakes that are suitable for invader predators like the Amazonian peacock bass tucunaré (*Cichla* spp.) [54, 55]. Biological invasions also followed the intensification of intentional and unintentional introductions due to aquaculture demands [56, 57] and fish stocking policies in the floodplain [29] and its surrounding reservoirs [58, 59]. In addition, interbreeding native and non-native species were introduced by fish stocking policies [60], and native species were raised in fish farms [56], which reinforce the hypothesis of weak or lacking resistance of natives to non-natives and could explain the dominance of a few species in the species pool in the most recent sampling periods.

Indeed, the greater increase of non-natives in gillnet samples compared to seines could be result of mass effects [61] particularly after flooding periods. Functional characteristics and activity levels of non-native invaders are frequently high [37, 54], and those individuals could be easily caught by gillnets [61]. In addition, fish occurrences were often associated with aquatic macrophytes in lacustrine and ecologically structured environments, this is, gillnet data is more precise and might present a more accurate picture of fish communities at the studied area [62]. Large and diverse macrophyte stands increased the niche breadth of the non-native fish such as *Moenkhausia forestii* Benine, Mariguela, Oliveira, 2009 [63]. Some of these macrophytes were also non-native, for example, *Hydrilla verticillata* (L.f.) Royle, which served as a new habitat for native and non-native fish [62] in an example of co-introduction. Environmental structuring created new habitat for several native and non-native species associated with the presence of macrophytes in the lacustrine environment of the studied floodplain [64]. Therefore, in agreement with previous studies [37], it is possible that biotic acceptance occurred in these already rich assemblages both in lakes and ‘rbai’.

Accordingly, rivers were the most obvious route between the sources of propagules (nearby fish farms and large reservoirs), and the lakes. In association with strong rainfall, that occurred during the intermediate (2010 and 2011), and final periods of the time series (2015 and 2016), non-native fish introductions and spillover in lakes [38] increased on affluents of the Paraná River floodplain [56]. The water released from upstream reservoirs also increased the landscape connectivity across aquatic environments and the community similarity [39]. Following flood in 2010 and 2011 [34], non-natives overwhelmed the native biodiversity *Q* for the first time, and the non-native biodiversity grew while native assemblages homogenised in the following years. The presence and abundance of alien species increased steeply in the lakes and rivers, especially after flooding periods, and the contamination indices reflected fish introductions especially in lakes ’lgar’ and ’lgua’, the most susceptible to the occurrence of alien species.

Many factors account for the success of the non-native species, such the lack of biotic resistance to non-native individuals [65], functional differences [66], and adaptive advantages [37]. Nevertheless, further studies on specific biotic interactions are needed to infer cause-effect relationships between natives and non-native species, by considering also trophic competition and interactions, after the flooding of the the Sete Quedas weterfalls. Instead, we confirmed that the way of introduction, natural flooding dynamics, and the species introduced may be important factors influencing individual survival in the new environment [37]. A recent study of aquarium fish farms showed that colonisation pressure of fish communities escaping from aquarium tanks was high in surrounding streams, but many species did not establish [21]; therefore, establishment of non-native species may be less evident in streams immediately surrounding the source. Alternatively, the riverine system ‘rbai’ was shorter in length, with a lower flow velocity to size ration than ‘rivi’ and ‘rpar’, approaching a lacustrine environment susceptible to the establishment of non-native species, particularly following floods [38, 54].

Alternative hypothesis such as intra-guild competition and biological invasions [37], environmental variation favouring feeding strategies [34], and other external factors could explain the loss of native species. Native assemblages with higher species richness at the beginning of the time series consistently lost biodiversity through time, and the coexistence between native and non-native fish species through time reveal that dominant native individuals prospered but without apparent resistance to the non-native fish colonisation [67]. The temporal correlation between species richness and biomass was positive and increased both for non-native and native assemblages, such as for “rivi”, and the establishment of newly introduced species apparently occurs without “invasion resistance” [40]. In addition, successful invaders were relatively different, considering native functional traits, therefore increasing the probability of biotic acceptance and suggesting that non-native species at least partially increased the overall realized niche in an heterogeneous space [66].

Even if the Upper Paraná River floodplain has been well investigated [20, 28, 31, 39, 66, 68], to our knowledge, the assessment of native and non-native fish biodiversity through species richness, biomass, their correlation, and Kempton’s *Q* were tested for the first time at each lake and river that had been monitored regularly from 2000 to 2017. Through the application of these simple and replicable indices of contamination and propagule pressure, we demonstrate important ecological processes and the long-term functioning of fish assemblages in the investigated Neotropical floodplain. We captured the turnover point where fish assemblages constituted by non-native species outweighed the biodiversity of native assemblages after 2010, which encourage additional effort for long-term monitoring and analyses accounting for the influence of non-native populations in each of the investigated habitats. Our analyses reinforce the need for the continuation of the research monitoring program and preservation of this undammed and pristine fluvial watercourse [69]. An approach to biotic interactions or food web models could reveal further local dynamics associated with interactions between native and non-native species. Based on our approach, decision makers could now target the worst scenarios of biological invasions, such as in ‘rbai’ and lakes.

The loss of species is a global tragedy [16], and demands the study particularly of rare species with important structuring roles and unique functionalities in the ecosystem [70]. Because of the intense efforts of scientists and environmental managers, the Paraná River floodplain still preserves most of the native species’ pool, which can be a good indicator of the efficiency of the surrounding preserved areas. Most species are small sized [71] and their survivorship can be crucial to the development of ecosystem services, and human populations [72]. However, local extinctions were not rare events during this investigation. In addition, some of the allegedly protected areas (i.e., at the Ivinhema River basin) presented a steeper increase of non-native species than non-protected environments, which could be a result of the relaxation of historical protection measures.

We conclude that the native fish biodiversity in the investigated environments suffered biotic homogenisation and non-native diversification under influence of non-native fish establishment, which resulted in the predominance of non-native biomass at the end of the time series in the upper Parana River floodplain. The ongoing predominance of non-native over native biodiversity has occurred in recent years at the expense of decreasing native biodiversity, particularly in floodplain lakes. This artificial state of the fish community can be reversible, even if biotic resistance by native fish assemblages does not prevent biological invasions. To smooth the effects of non-native assemblage, restoration programs are encouraged particularly in the most contaminated environments.

## Supporting information

S1 Table Gillnet set used at each locality in the long-term monitoring of lakes and rivers in the upper Parana River floodplain (2000 to 2017). Number (N).
(DOCX)

S2 Table List of species captured by gillnets and seines in three rivers and six lagoons of the upper Parana River floodplain, from 2000 to 2017 [1]. Species names are alphabetically ordered and classified according to the species origin (native or non-native of the upper Parana River section). Some groups which could include native and non-native species were not classified (NA).
(DOCX)

S1 Fig Spearman’s correlation coefficient (rho) and Kempton’s Index (Q) between biomass (g.m-2, gillnetting and beach seining in lakes) and species richness of native and non-native fish at six lakes and three riverine channels of the Paraná River floodplain sampled seasonally by gillnets’ from 2000 to 2017. The term “ns” stands for non-significative differences.
(DOCX)
