# Supplementary material for: Long-term contamination by non-native fish assemblages in a Neotropical floodplain
Source: PLoS One. 2024 Nov 11;19(11):e0311018. doi: 10.1371/journal.pone.0311018 (PMC11554207; doi:10.1371/journal.pone.0311018)
Supplement: S1 Table — Number (N). (DOCX) [file pone.0311018.s001.docx]

Supplementary Material

**Long-term contamination by non-native fish assemblages in a Neotropical floodplain**

Short: Fish biodiversity trends in the upper Parana River floodplain

Luis Artur Valões Bezerra (ORCID 0000-0003-1954-5556)^1*^, Simone Libralato^2^, Jan Kubečka^1^, Andre Andrian Padial^3,4^

^1^Institute of Hydrobiology, Biology Centre of the Czech Academy of Sciences (BC-CAS), České Budejovice, Czechia.

*Corresponding author, email: larturr@yahoo.com.br

^2^National Institute of Oceanography and Applied Geophysics - OGS, Trieste, Italy.

^3^Laboratorio de Análise e Síntese em Biodiversidade (LASB), Departamento de Botânica, Programa de Pós-graduação em Ecologia e Conservação (PPGECO-UFPR) and Programa de Pós-graduação em Botânica, Universidade Federal do Paraná, Curitiba, Brazil.

^4^Programa de Pós-graduação em Ecologia de Ambientes Aquáticos Continentais, Núcleo de Pesquisa em Limnologia, Ictiologia e Aquicultura (NUPELIA), Universidade Estadual de Maringá, Maringá, Brazil.

Authors: Luis Artur Valões Bezerra, Simone Libralato, Jan Kubečka, and Andre Andrian Padial

| **S1 Table**. Gillnet set used at each locality in the long-term monitoring of lakes and rivers in the upper Parana River floodplain (2000 to 2017). Number (N). | | | | |
| --- | --- | --- | --- | --- |
| Mesh (cm) | Height (m) | Length (m) | N. of Nodes | Area (m^2^) |
| 2.4 | 2.4 | 20 | 12 | 48 |
| 3 | 1.5 | 20 | 15 | 30 |
| 4 | 1.5 | 20 | 20 | 30 |
| 5 | 1.5 | 20 | 25 | 30 |
| 6 | 1.5 | 20 | 30 | 30 |
| 7 | 1.75 | 20 | 35 | 35 |
| 8 | 1.6 | 20 | 40 | 32 |
| 10 | 1.7 | 20 | 50 | 34 |
| 12 | 1.55 | 20 | 60 | 31 |
| 14 | 1.7 | 20 | 70 | 34 |
| 16 | 1.7 | 20 | 80 | 34 |
