# Supplementary material for: Long-term contamination by non-native fish assemblages in a Neotropical floodplain
Source: PLoS One. 2024 Nov 11;19(11):e0311018. doi: 10.1371/journal.pone.0311018 (PMC11554207; doi:10.1371/journal.pone.0311018)
Supplement: S2 Table — Species names are alphabetically ordered and classified according to the species origin (native or non-native of the upper Parana River section). Some groups which could include native and non-native species were not classified (NA). (DOCX) [file pone.0311018.s002.docx]

Supplementary Material

**Long-term contamination by non-native fish assemblages in a Neotropical floodplain**

Short: Fish biodiversity trends in the upper Parana River floodplain

Luis Artur Valões Bezerra (ORCID 0000-0003-1954-5556)^1*^, Simone Libralato^2^, Jan Kubečka^1^, Andre Andrian Padial^3,4^

^1^Institute of Hydrobiology, Biology Centre of the Czech Academy of Sciences (BC-CAS), České Budejovice, Czechia.

*Corresponding author, email: larturr@yahoo.com.br

^2^National Institute of Oceanography and Applied Geophysics - OGS, Trieste, Italy.

^3^Laboratorio de Análise e Síntese em Biodiversidade (LASB), Departamento de Botânica, Programa de Pós-graduação em Ecologia e Conservação (PPGECO-UFPR) and Programa de Pós-graduação em Botânica, Universidade Federal do Paraná, Curitiba, Brazil.

^4^Programa de Pós-graduação em Ecologia de Ambientes Aquáticos Continentais, Núcleo de Pesquisa em Limnologia, Ictiologia e Aquicultura (NUPELIA), Universidade Estadual de Maringá, Maringá, Brazil.

Authors: Luis Artur Valões Bezerra, Simone Libralato, Jan Kubečka, and Andre Andrian Padial

**S2 Table**. List of species captured by gillnets and seines in three rivers and six lagoons of the upper Parana River floodplain, from 2000 to 2017. Species names are alphabetically ordered and classified according to the species origin (native or non-native of the upper Parana River section). Some groups which could include native and non-native species were not classified (NA).

| Species | Author | Origin |
| --- | --- | --- |
| *Acestrorhynchus lacustris* | (Lütken, 1875) | Native |
| *Acestrorhynchus pantaneiro* | Menezes, 1992 | Non- native |
| *Aequidens plagiozonatus* | Kullander, 1984 | Non- native |
| *Ageneiosus inermis* | (Linnaeus, 1766) | Non- native |
| *Ageneiosus militaris* | Valenciennes, 1835 | Native |
| *Ageneiosus ucayalensis* | Castelnau, 1855 | Non- native |
| *Apareiodon affinis* | (Steindachner, 1879) | Native |
| *Aphyocharax anisitsi* | Eigenmann & Kennedy, 1903 | Native |
| *Aphyocharax dentatus* | Eigenmann & Kennedy, 1903 | Non- native |
| *Aphyocheirodon hemigrammus* | Eigenmann, 1915 | Native |
| *Apistogramma commbrae* | (Regan, 1906) | Non- native |
| *Apteronotus caudimaculosus* | de Santana, 2003 | Non- native |
| *Apteronotus ellisi* | (Alonso de Arámburu, 1957) | Native |
| *Astronotus crassipinnis* | (Heckel, 1840) | Non- native |
| *Astyanax lacustris* | (Lütken, 1875) | Native |
| *Auchenipterus osteomystax* | (Miranda Ribeiro, 1918) | Non- native |
| *Brachyhypopomus gauderio* | Giora & Malabarba, 2009 | Non- native |
| *Brycon hilarii* | (Valenciennes, 1850) | Non- native |
| *Brycon orbignyanus* | (Valenciennes, 1850) | Native |
| *Callichthys callichthys* | (Linnaeus, 1758) | Native |
| *Catathyridium jenynsii* | (Günther, 1862) | Non- native |
| *Characidium* sp. |  | Native |
| *Characidium zebra* | Eigenmann, 1909 | Native |
| *Cichla kelberi* | Kullander & Ferreira, 2006 | Non- native |
| *Cichla monoculus* | Agassiz, 1831 | Non- native |
| *Cichla piquiti* | Kullander & Ferreira, 2006 | Non- native |
| *Cichlasoma paranaense* | Kullander, 1983 | Native |
| *Clarias gariepinus* | (Burchell, 1822) | Non- native |
| *Colossoma macropomum* | (Cuvier, 1816) | Non- native |
| *Crenicichla britskii* | Kullander, 1982 | Native |
| *Crenicichla haroldoi* | Luengo & Britski, 1974 | Native |
| *Crenicichla jaguarensis* | Haseman, 1911 | Native |
| *Crenicichla* sp. |  | Native |
| *Cyphocharax modestus* | (Fernández-Yépez, 1948) | Native |
| *Cyphocharax nagelii* | (Steindachner, 1881) | Native |
| *Diapoma guarani* | (Mahnert, Géry, 1987) | Native |
| *Eigenmannia trilineata* | López & Castello, 1966 | Native |
| *Eigenmannia virescens* | (Valenciennes, 1836) | Native |
| *Erythrinus erythrinus* | (Bloch & Schneider, 1801) | Non- native |
| *Farlowella hahni* | Meinken, 1937 | Non- native |
| *Galeocharax gulo* | (Cope, 1870) | Native |
| *Geophagus iporangensis* | Haseman, 1911 | Native |
| *Geophagus sveni* | Lucinda, Lucena & Assis, 2010 | Non- native |
| *Gymnorhamphichthys britskii* | Carvalho, Ramos & Albert, 2011 | Non- native |
| *Gymnotus inaequilabiatus* | (Valenciennes, 1839) | Native |
| *Gymnotus pantanal* | Fernandes, Albert, Daniel-Silva, Lopes, Crampton & Almeida-Toledo, 2005 | Non- native |
| *Gymnotus paraguensis* | Albert & Crampton, 2003 | Non- native |
| *Gymnotus sylvius* | Albert & Fernandes-Matioli, 1999 | Native |
| *Hemigrammus ora* | Zarske, Le Bail & Géry, 2006 | Non- native |
| *Hemiodus orthonops* | Eigenmann & Kennedy, 1903 | Non- native |
| *Hemisorubim platyrhynchos* | (Valenciennes, 1840) | Native |
| *Hoplerythrinus unitaeniatus* | (Spix & Agassiz, 1829) | Non- native |
| *Hoplias intermedius* | (Günther, 1864) | Native |
| *Hoplias malabaricus* | (Bloch, 1794) | Native |
| *Hoplias malabaricus* | (Bloch, 1794) | Native |
| *Hoplias mbigua* | Azpelicueta, Benítez, Aichino, Mendez,2015 | Non- native |
| *Hoplosternum littorale* | (Hancock, 1828) | Native |
| Hybrid |  | Non- native |
| *Hyphessobrycon eques* | (Steindachner, 1882) | Non- native |
| *Hypophthalmus oremaculatus* | Nani & Fuster, 1947 | Non- native |
| *Hypostomus albopunctatus* | (Regan, 1908) | Native |
| *Hypostomus ancistroides* | (Ihering, 1911) | Native |
| *Hypostomus cochliodon* | Kner, 1854 | Non- native |
| *Hypostomus commersoni* | Valenciennes, 1836 | Non- native |
| *Hypostomus hermanni* | (Ihering, 1905) | Native |
| *Hypostomus iheringii* | (Regan, 1908) | Native |
| *Hypostomus microstomus* | Weber, 1987 | Non- native |
| *Hypostomus regani* | (Ihering, 1905) | Native |
| *Hypostomus strigaticeps* | (Regan, 1908) | Native |
| *Hypostomus ternetzi* | (Boulenger, 1895) | Non- native |
| *Iheringichthys labrosus* | (Lütken, 1874) | Non- native |
| *Knodus moenkhausii* | (Eigenmann & Kennedy, 1903) | Non- native |
| *Laetacara araguaiae* | Ottoni & Costa, 2009 | Non- native |
| *Leporellus vittatus* | (Valenciennes, 1850) | Native |
| *Leporinus friderici* | (Bloch, 1794) | Native |
| *Leporinus lacustris* | Amaral Campos, 1945 | Native |
| *Leporinus octofasciatus* | Steindachner, 1915 | Native |
| *Leporinus striatus* | Kner, 1858 | Native |
| *Leporinus tigrinus* | Borodin, 1929 | Non- native |
| *Lepthoplosternum pectorale* | (Boulenger, 1895) | Non- native |
| *Loricaria* sp. |  | Native |
| *Loricariichthys platymetopon* | Isbrücker & Nijssen, 1979 | Non- native |
| *Loricariichthys rostratus* | Reis & Pereira, 2000 | Non- native |
| *Megalancistrus parananus* | (Peters, 1881) | Native |
| *Megaleporinus macrocephalus* | (Garavello & Britski, 1988) | Non- native |
| *Megaleporinus obtusidens* | (Valenciennes, 1837) | Native |
| *Megaleporinus piavussu* | Britski, Birindelli & Garavello, 2012 | Native |
| *Megalonema platanum* | (Günther, 1880) | Native |
| *Melanorivulus* sp. |  | Native |
| *Metynnis lippincottianus* | (Cope, 1870) | Non- native |
| *Moenkhausia australe* | Eigenmann, 1908 | Non- native |
| *Moenkhausia bonita* | Benine, Castro & Sabino, 2004 | Native |
| *Moenkhausia forestii* | Benine, Mariguela & Oliveira, 2009 | Non- native |
| *Moenkhausia gracilima* | Eigenmann, 1908 | Native |
| *Moenkhausia intermedia* | Eigenmann, 1908 | Native |
| *Myloplus tiete* | (Eigenmann & Norris, 1900) | Native |
| *Odontostilbe avanhandava* | Chuctaya, Bührnheim, Malabarba, 2018 | Native |
| *Oligosarcus paranensis* | Menezes & Géry, 1983 | Native |
| *Oligosarcus pintoi* | Amaral Campos, 1945 | Native |
| *Ossancora eigenmanni* | (Boulenger, 1895) | Non- native |
| *Pamphorichthys hollandi* | (Henn, 1916) | Native |
| *Parauchenipterus galeatus* | (Linnaeus, 1766) | Non- native |
| *Parodon nasus* | Kner, 1859 | Native |
| *Piabarchus stramineus* | (Eigenmann, 1908) | Native |
| *Piaractus mesopotamicus* | (Holmberg, 1887) | Native |
| *Pimelodella avanhandavae* | Eigenmann, 1917 | Native |
| *Pimelodella gracilis* | (Valenciennes, 1835) | Native |
| *Pimelodella taenioptera* | Miranda Ribeiro, 1914 | Non- native |
| *Pimelodus microstoma* | Steindachner, 1877 | Native |
| *Pimelodus mysteriosus* | Azpelicueta, 1998 | Native |
| *Pimelodus ornatus* | Kner, 1858 | Non- native |
| *Pimelodus* sp |  | NA |
| *Pinirampus pirinampu* | (Spix & Agassiz, 1829) | Native |
| *Plagioscion squamosissimus* | (Heckel, 1840) | Non- native |
| *Platanichthys platana* | (Regan, 1917) | Non- native |
| *Platydoras armatulus* | (Valenciennes, 1840) | Non- native |
| *Poecilia reticulata* | Peters, 1859 | Non- native |
| *Potamotrygon amandae* | Loboda & Carvalho, 2013 | Non- native |
| *Potamotrygon falkneri* | Castex & Maciel, 1963 | Non- native |
| *Prochilodus lineatus* | (Valenciennes, 1837) | Native |
| *Proloricaria prolixa* | (Isbrücker & Nijssen, 1978) | Native |
| *Psalidodon* aff. *fasciatus* | (Cuvier, 1819) | Native |
| *Psalidodon* aff. *paranae* | Eigenmann, 1914 | Native |
| *Psalidodon schubarti* | Britski, 1964 | Native |
| *Psellogrammus kennedyi* | (Eigenmann, 1903) | Non- native |
| *Pseudopimelodus mangurus* | (Valenciennes, 1835) | Native |
| *Pseudoplatystoma corruscans* | (Spix & Agassiz, 1829) | Native |
| *Pterodoras granulosus* | (Valenciennes, 1821) | Non- native |
| *Pterygoplichthys ambrosettii* | (Holmberg, 1893) | Non- native |
| *Pyrrhulina australis* | Eigenmann & Kennedy, 1903 | Native |
| *Rhamdia quelen* | (Quoy & Gaimard, 1824) | Native |
| *Rhamphichthys hahni* | (Meinken, 1937) | Non- native |
| *Rhaphiodon vulpinus* | Spix & Agassiz, 1829 | Native |
| *Rhinelepis aspera* | Spix & Agassiz, 1829 | Native |
| *Rhinodoras dorbignyi* | (Kner, 1855) | Native |
| *Roeboides descalvadensis* | Fowler, 1932 | Non- native |
| *Salminus brasiliensis* | (Cuvier, 1816) | Native |
| *Salminus hilarii* | Valenciennes, 1850 | Native |
| *Satanoperca* sp. |  | Native |
| *Schizodon altoparanae* | Garavello & Britski, 1990 | Native |
| *Schizodon borellii* | (Boulenger, 1900) | Non- native |
| *Schizodon nasutus* | Kner, 1858 | Native |
| *Serrapinnus calliurus* | (Boulenger, 1900) | Non- native |
| *Serrapinnus heterodon* | (Eigenmann, 1915) | Native |
| *Serrapinnus notomelas* | (Eigenmann, 1915) | Native |
| *Serrapinnus* spp. |  | NA |
| *Serrasalmus maculatus* | Kner, 1858 | Native |
| *Serrasalmus marginatus* | Valenciennes, 1837 | Non- native |
| *Sorubim lima* | (Bloch & Schneider, 1801) | Non- native |
| *Steindachnerina brevipinna* | (Eigenmann & Eigenmann, 1889) | Non- native |
| *Steindachnerina insculpta* | (Fernández-Yépez, 1948) | Native |
| *Sternopygus macrurus* | (Bloch & Schneider, 1801) | Native |
| *Synbranchus marmoratus* | Bloch, 1795 | Native |
| *Trachydoras paraguayensis* | (Eigenmann & Ward, 1907) | Non- native |
| *Zungaro jahu* | (Ihering, 1898) | Native |
