# Supplementary material for: Long-term contamination by non-native fish assemblages in a Neotropical floodplain
Source: PLoS One. 2024 Nov 11;19(11):e0311018. doi: 10.1371/journal.pone.0311018 (PMC11554207; doi:10.1371/journal.pone.0311018)
Supplement: S1 Fig — The term “ns” stands for non-significative differences. (DOCX) [file pone.0311018.s003.docx]

Supplementary Material

**Long-term contamination by non-native fish assemblages in a Neotropical floodplain**

Short: Fish biodiversity trends in the upper Parana River floodplain

Luis Artur Valões Bezerra (ORCID 0000-0003-1954-5556)^1*^, Simone Libralato^2^, Jan Kubečka^1^, Andre Andrian Padial^3,4^

^1^Institute of Hydrobiology, Biology Centre of the Czech Academy of Sciences (BC-CAS), České Budejovice, Czechia.

*Corresponding author, email: larturr@yahoo.com.br

^2^National Institute of Oceanography and Applied Geophysics - OGS, Trieste, Italy.

^3^Laboratorio de Análise e Síntese em Biodiversidade (LASB), Departamento de Botânica, Programa de Pós-graduação em Ecologia e Conservação (PPGECO-UFPR) and Programa de Pós-graduação em Botânica, Universidade Federal do Paraná, Curitiba, Brazil.

^4^Programa de Pós-graduação em Ecologia de Ambientes Aquáticos Continentais, Núcleo de Pesquisa em Limnologia, Ictiologia e Aquicultura (NUPELIA), Universidade Estadual de Maringá, Maringá, Brazil.

Authors: Luis Artur Valões Bezerra, Simone Libralato, Jan Kubečka, and Andre Andrian Padial


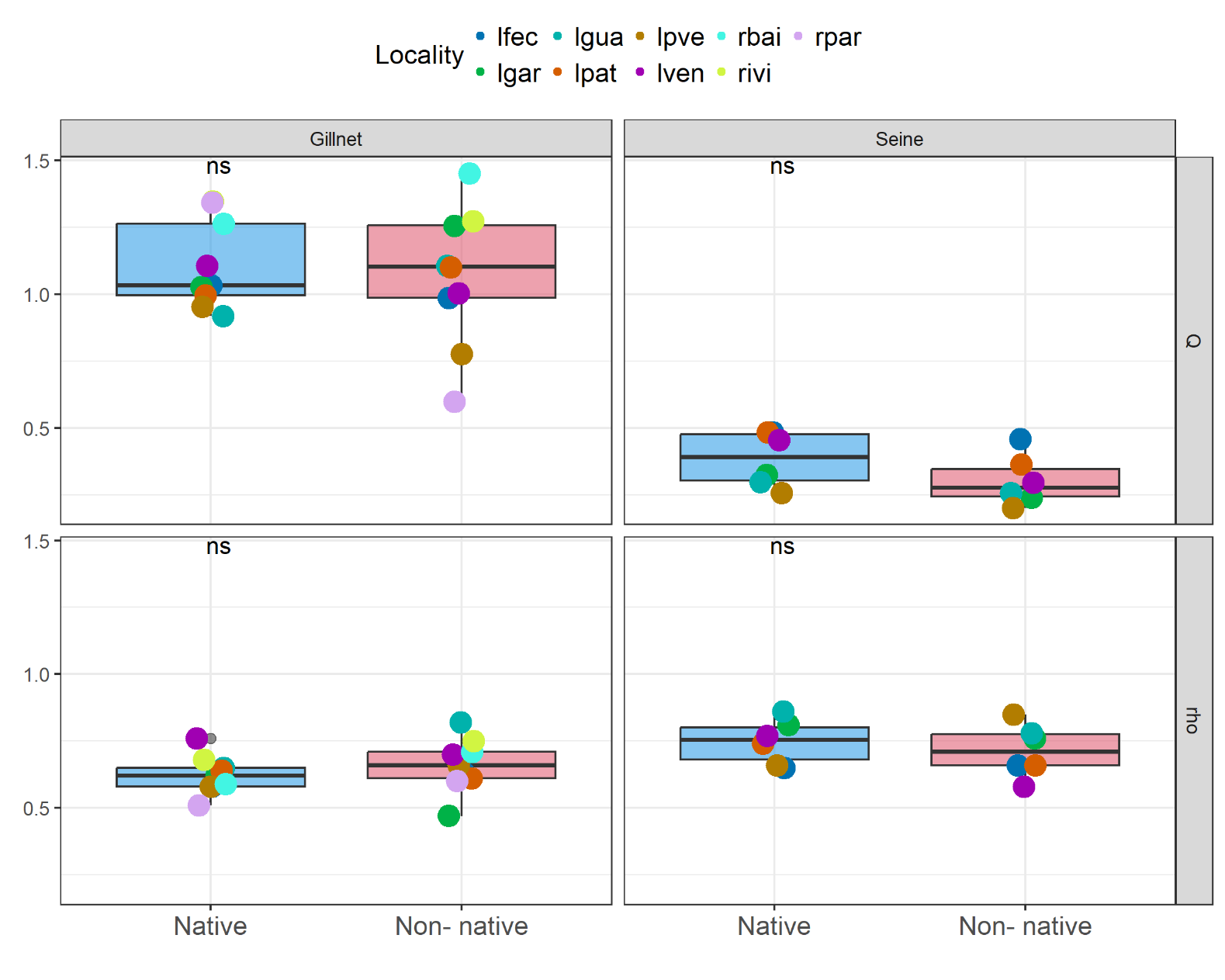


**S1 Figure**. Spearman’s correlation coefficient (rho) and Kempton’s Index (Q) between biomass (g.m^-2^, gillnetting and beach seining in lakes) and species richness of native and non-native fish at six lakes and three riverine channels of the Paraná River floodplain sampled seasonally by gillnets’ from 2000 to 2017. The term “ns” stands for non-significative differences.
